# Trajectories of alcohol consumption during life and the risk of developing breast cancer

**DOI:** 10.1038/s41416-021-01492-w

**Published:** 2021-09-06

**Authors:** Carolina Donat-Vargas, Ángel Guerrero-Zotano, Ana Casas, José Manuel Baena-Cañada, Virginia Lope, Silvia Antolín, José Ángel Garcia-Saénz, Begoña Bermejo, Montserrat Muñoz, Manuel Ramos, Ana de Juan, Carlos Jara Sánchez, Pedro Sánchez-Rovira, Antonio Antón, Joan Brunet, Joaquín Gavilá, Javier Salvador, Esperanza Arriola Arellano, Susana Bezares, Nerea Fernández de Larrea-Baz, Beatriz Pérez-Gómez, Miguel Martín, Marina Pollán

**Affiliations:** 1 IMDEA-Food Institute, CEI UAM+CSIC, Madrid, Spain; 2 Department of Preventive Medicine and Public Health, School of Medicine, Universidad Autónoma de Madrid-IdiPaz, CIBERESP (CIBER of Epidemiology and Public Health), Madrid, Spain; 3 Unit of Cardiovascular and Nutritional Epidemiology, Institute of Environmental Medicine, Karolinska Institutet, Stockholm, Sweden; 4 Medical Oncology Unit, Instituto Valenciano de Oncología, Valencia, Spain; 5 Medical Oncology Unit, Hospital Virgen del Rocío, Sevilla, Spain; 6 Medical Oncology Unit, Hospital Puerta del Mar, Cádiz, Spain; 7 Instituto de Investigación en Biomedicina de Cádiz (INiBICA), Cádiz, Spain; 8 National Center for Epidemiology, Carlos III Institute of Health, Madrid, Spain; 9 Consortium for Biomedical Research in Epidemiology and Public Health (CIBERESP), Institute of Health Carlos III, Madrid, Spain; 10 Medical Oncology Unit, Complejo Hospitalario Universitario, A Coruña, Spain; 11 Medical Oncology Unit, Hospital Clínico Universitario San Carlos, Madrid, Spain; 12 Medical Oncology Unit, Hospital Clínico, Valencia, Spain; 13 Medical Oncology Unit, Hospital Clinic i Provincial, Barcelona, Spain; 14 Translational Genomics and Targeted Therapeutics, Institut d’Investigacions Biomèdiques Pi i Sunyer-IDIBAPS, Barcelona, Spain; 15 Medical Oncology Unit, Centro Oncológico de Galicia, A Coruña, Spain; 16 Medical Oncology Unit, Hospital Marqués de Valdecilla, Santander, Spain; 17 Medical Oncology Unit/Departamento Especialidades Médicas, Hospital Universitario Fundación Alcorcón/Universidad Rey Juan Carlos, Madrid, Spain; 18 Medical Oncology Unit, Hospital Universitario de Jaén, Jaén, España; 19 Medical Oncology Unit, Hospital Universitario Miguel Servet, Zaragoza, España; 20 Medical Oncology Department, Institut Català d’Oncologia, IDIBGi, Girona, Spain; 21 Medical Sciences Department, Universitat de Girona, Girona, Spain; 22 GEICAM Spanish Breast Cancer Research Group, Madrid, Spain; 23 Medical Oncology Unit, Instituto de Investigación Sanitaria Gregorio Marañón/Universidad Complutense, Madrid, Spain; 24 Centro de Investigación Biomédica en Red de Oncología, CIBERONC-ISCIII, Madrid, Spain

**Keywords:** Risk factors, Epidemiology

## Abstract

**Background:**

Whether there are lifetime points of greater sensitivity to the deleterious effects of alcohol intake on the breasts remains inconclusive.

**Objective:**

To compare the influence of distinctive trajectories of alcohol consumption throughout a woman’s life on development of breast cancer (BC).

**Methods:**

1278 confirmed invasive BC cases and matched (by age and residence) controls from the Epi-GEICAM study (Spain) were used. The novel group-based trajectory modelling was used to identify different alcohol consumption trajectories throughout women’s lifetime.

**Results:**

Four alcohol trajectories were identified. The first comprised women (45%) with low alcohol consumption (<5 g/day) throughout their life. The second included those (33%) who gradually moved from a low alcohol consumption in adolescence to a moderate in adulthood (5 to <15 g/day), never having a high consumption; and oppositely, women in the third trajectory (16%) moved from moderate consumption in adolescence, to a lower consumption in adulthood. Women in the fourth (6%) moved from a moderate alcohol consumption in adolescence to the highest consumption in adulthood (≥15 g/day), never having a low alcohol consumption. Comparing with the first trajectory, the fourth doubled BC risk (OR 2.19; 95% CI 1.27, 3.77), followed by the third (OR 1.44; 0.96, 2.16) and ultimately by the second trajectory (OR 1.17; 0.86, 1.58). The magnitude of BC risk was greater in postmenopausal women, especially in those with underweight or normal weight. When alcohol consumption was independently examined at each life stage, ≥15 g/day of alcohol consumption in adolescence was strongly associated with BC risk followed by consumption in adulthood.

**Conclusions:**

The greater the alcohol consumption accumulated throughout life, the greater the risk of BC, especially in postmenopausal women. Alcohol consumption during adolescence may particularly influence BC risk.

## Introduction

Breast cancer (BC) remains the most frequently diagnosed cancer in women in the majority of the countries worldwide, abruptly disrupting the lives of millions of women [1]. Amongst the aetiological factors proposed to be implicated in developing BC, reproductive history has been one of the most consistent [2]. Likewise, alcohol consumption is the only significant and consistently clinically supported single dietary risk factor for BC [3–5]. Although a linear association between alcohol consumption and BC is firmly established [5], whether alcohol consumption acts early in the process of breast tumorigenesis and whether there are time points in the lifespan of greater sensitivity to the deleterious effects of ethanol on women’s breasts remain unanswered.

Compared with other organs, the breast appears to be more susceptible to the carcinogenic effects of alcohol, particularly from menarche to the first pregnancy, since the mammary glands are not completely differentiated [6–8]. Human data supports that exposures before the first pregnancy may be more critical in BC development than exposures later in life [8–11].

Nonetheless, previous epidemiological studies on BC addressing alcohol consumption at different periods throughout life have generated inconsistent results, and the risk attributable to alcohol consumed during young ages remains uncertain [12]. To improve upon past research and to afford a most comprehensive evaluation of the effect of alcohol consumption throughout a woman’s life, we aimed to chart out different life trajectories of alcohol consumption using a novel methodology, the Group-Based Trajectory Modeling (GBTM), and compare them in terms of the risk of BC, overall, by menopausal status and by pathologic subtype. This novel statistical method identifies clusters of women who followed similar patterns of alcohol consumption over time. This approach has the advantage over traditional analysis of capturing the life course alcohol consumption [13, 14] rather than cumulative consumption over time or single consumptions at different time periods (without considering the impact of alcohol consumption during any other period). To our knowledge, this the first study to evaluate trajectories of alcohol consumption over the life in relation to BC risk.

## Methods

### Study design and population

Present data come from the EpiGEICAM study, a multicenter 1:1 matched case–control study on female BC. To be eligible for the study, participants had to reside in one of the hospitals´ catchment area, be between 18–70 years old and be able to complete the epidemiological questionnaire. The global participation rate was 82% (75% in cases and 90% in controls).

Thus, 1017 women newly diagnosed with BC, and histologically confirmed, were recruited, between 2006 and 2011, in the Oncology Departments of 23 hospitals which are members of the Spanish Breast Cancer Research Group, GEICAM (http://www.geicam.org/). These hospitals are situated in 9 Spanish Autonomous Regions, accounting for 78% of the Spanish population. Each BC case was matched with a healthy control residing in the same town and of similar age (±5 years), generally selected by the case (non-blood relatives, friends, neighbours, work colleagues). Patients´ blood-relatives were not eligible. Cases were subclassified based on tumour characteristics according to expression of oestrogen receptor (ER), progesterone receptor (PR), and luminal human epidermal growth factor receptor 2 (HER2):(i) HER2 negative tumours (ER+ or PR+ with HER2–); (ii) HER2 positive tumours (HER2+ irrespective of ER or PR status); and (iii) triple-negative tumours (ER–, PR– and HER2–) [15–17].

### Data collection

Cases and controls filled a structured questionnaire which recorded demographic and anthropometric data, personal and family background, medical and occupational history and lifestyle and dietary information. Cases completed the questionnaire within 3 months after diagnosis of BC. When questions referred to a specific time period for cases, the corresponding control was asked about the same calendar period.

Data collected for each participant included age, educational level, height and weight, personal medical history (with special emphasis on obstetric and gynaecological background) including menopausal status, age at menarche, number of children, age at first birth, use of hormone replacement therapy (HRT), and previous diagnosis of benign breast diseases; occupational history, family history of BC, physical activity during the previous year, smoking status, and diet. Based on the highest educational level achieved, participants were classified into 3 categories: primary school or less, secondary school, and university graduate or higher. Body mass index (BMI) was calculated based on participant’s self-reported weight one year before the interview and height. Postmenopausal status was defined as the absence of menstruation in the last 12 months. Physical activity during the previous year was collected through a detailed questionnaire and recreational, occupational, and household activities were considered to classifying women using 3 categories: sedentary/ lightly active, moderately and active/very active [18, 19].

Dietary intake in the past 5 years was measured using a 117-item semiquantitative food frequency questionnaire (FFQ), similar to the Harvard questionnaire [20], adapted to and validated in different Spanish adult populations [21, 22]. Total intake of each nutrient and of total energy was computed for each participant. Adherence to a Mediterranean dietary pattern, previously identified from the control group, was calculated [23]. The questionnaire was self-fulfilled and then jointly reviewed by the participant and a trained interviewer in each centre. Data entry and quality control were performed at the GEICAM headquarters. The accuracy of the information registered in the database was verified randomly selecting and reviewing 10% of the questionnaires.

### Alcohol consumption assessment

The FFQ had a specific module for the detailed measurement of the consumption of different alcoholic beverages. Participants had nine options to describe how frequently they consumed each type of beverage –ranging from ‘never or less than once per month’ to ‘six or more times per day’—at specific life stages —adolescence (12–19 years), young adulthood (20–29 years) and adulthood (in the recent 5-year period). The different drinks included were wine (red, white, rosé), vermouth-type alcohols (sherry, dry wines), beer, cider, cava and high-grade distillated spirits (40°, such as brandy, gin, rum, whiskey, vodka and tequila). Responses were converted to mean daily grams (g) of alcohol consumed by multiplying consumption frequency by the corresponding typical ethanol content (g) of each alcoholic beverage [24] and its standard serving sizes specified in the FFQ (e.g. the serving size of red wine is 125 cc, while the serving size of beer is 200 cc and for liquor it is 50 cc). Finally, consumption of each type of alcoholic beverage and consumption of total alcohol (g/day) were computed for each participant and for each of the three life stages. Total alcohol consumption was categorised in low, moderate and high according to previous published cut off points: <5, 5–<15 and ≥15 g/day [25, 26].

### Statistical methods

In the group-based trajectory modelling (GBTM), a group is conceptually thought of as a latent longitudinal stratum where population variability is captured by differences across groups in the shape and level of their trajectories. This method fits longitudinal data as a discrete mixture of two or more trajectories via maximum likelihood [27]. In this study, the GBTM was used to identify different alcohol consumption trajectories throughout the life of those women with alcohol consumption data (categorised into low, moderate and high according to the cut off points: <5, 5–<15 and ≥15 g/day) in each of the life stages: adolescence (12–19 yr.), young adulthood (20–29 yr.) and adulthood (≥30 yr.).

To fit the models, we used the Stata traj plug-in, and total alcohol consumption (g/day) modelled as a censored normal distribution and as a polynomial function of age (as time scale). The model selection was carried out using an approach that consisted of two-stages assessing the change in the Bayesian Information Criterion (BIC) [28]. In the first stage, we determined the number of groups using a quadratic form for all the trajectory groups, and selected the model with the best BIC [28]. Once we identified that the model with four groups fit best, in the second stage, we aimed to determine the order of the polynomial functions (1 or 2) specifying the shape of each trajectory. The BIC indicated that the best model included the quadratic order term. This process was followed using the entire sample, as well as separately for cases and controls, consistently obtaining the same trajectory groups. The average posterior probability (APP) of an individual’s belonging to each of the trajectory groups was tested to verify the model´s adequacy. The APP of group membership measured the likelihood of each participant belonging to its assigned group. We used an APP ≥ 0.70 as the cutoff point [28]. Finally, to assess the assignment accuracy, we calculated the odds of correct classification (OCC) and consider that an OCC greater than 5.0 indicated that the model had a high assignment accuracy [28] (Table S1). These trajectories should be interpreted as groups of women following similar patterns of alcohol consumption across their life-course [29].

Participants’ characteristics were compared between cases and controls and across the four alcohol consumption trajectories, using counts and percentages for categorical variables and means and standard deviations for continuous variables. To check the statistical significance of case–control differences we used Pearson Chi-square test (for categorical variables), Student’s *t*-test (for continuous variables), and for variables with imputed values, *p* values resulting from logistic regression models.

To evaluate the association between life course alcohol consumption and BC, we first explored independently for each of the three life stages (adolescence, young adulthood, and adulthood), the association between total alcohol consumption (low, moderate, high) and the risk of BC. Next, as the main analysis, we assessed the association of the trajectories of alcohol identified by the GBTM with BC risk. For these analyses we fitted conditional logistic regression models, estimating odds ratios (ORs) and their 95% confidence intervals (CI)

Additionally, we performed analyses stratified by menopausal status and, among postmenopausal women, also by BMI (<25 and ≥25 kg/m^2^). For these analyses, we used unconditional logistic regression models. We further assessed the modifier effect by menopausal status and BMI by testing the interactions (likelihood-ratio test) between these variables and the trajectories of alcohol consumption.

To test whether the associations varied across pathological BC subtypes, we used multinomial logistic regression models with pathological subtype as the dependent variable and the control group as the reference (base outcome). OR (also referred to as relative risk ratios (RRRs) and their 95% CI were estimated and the heterogeneity of effects for the different BC subtypes was tested using the Wald statistic.

Among factors that could potentially confound the association between alcohol and BC, we included those covariates that changed the estimates by more than 5% in the main assessment. Thus, in addition to age and residence (matching variables), in the multivariate models we included body mass index (BMI) one year before entry in the study (<25, 25–<30, ≥30 kg/m^2^) and menopausal status (these two included with their corresponding interaction terms), educational level (primary school or less, secondary school, university graduate or higher), smoking status (never smoker, ex-smoker for ≥6 months and smoker or ex-smoker for <6 months), physical activity (sedentary/lightly active, moderately active, active/very active), total calories intake (continuous), level of adherence to the Mediterranean dietary pattern (quartiles based on controls), age at menarche (years), age at the first birth (years with a category of nulliparous), number of children (continuous), chronic diseases (yes/no), previous benign breast diseases (yes/no), family history of BC (yes/no), and hormone replacement therapy (HRT) use (yes/no).

Some variables contained missing values: BMI (9%), educational level (<1%), smoking (<0.5%), physical activity (7%), calories intake (3%), adherence to the Mediterranean dietary pattern (3%), age at menarche (0.6%), age at first birth (4%), previous benign breast lesions (2%), and HRT use (5%). In order to obtain unbiased estimates of the effect of each alcohol consumption trajectory using the information provided by all case–control pairs, missing values were imputed using multiple imputations with chained equations with 7 predictors (age, educational level, number of children, menopausal status, previous benign breast diseases, family history of BC and case/control status) and 5 imputations [30, 31]. The validity of the imputation was checked by comparing the results obtained with those resulting from the analyses of the data with complete information.

All *p* values presented are two-tailed; <0.05 was considered statistically significant. Analyses were carried out using STATA/SE version 16.0 (StataCorp, College Station, TX, USA).

## Results

Out of the 2034 recruited women (1017 case–control pairs), 1578 (799 controls and 779 cases) had complete longitudinal data on alcohol consumption (i.e. alcohol data in all three life stages). Among these women, 925 were premenopausal and 653 postmenopausal. 70.3% (*n* = 546) of the cases had a luminal tumour (ER+ and/or PR+ with HER2 negative), 18.5% (*n* = 144) had a HER2+ tumour and the remaining 11.2% (*n* = 87) had a TN (ER−, PR− and HER2) tumour (Table S2).

The average age was 49 (±9) years old for both cases and controls. On average, compared with controls, cases had a statistically significant lower educational level, lower proportion of postmenopausal women, had more frequently family history of breast cancer (first or second degree), a lower frequency of chronic diseases and slightly higher caloric intake (Table 1). Overall, no other meaningful differences in baseline characteristics were observed between cases and controls. Regarding alcohol consumption, on average, cases consumed more alcohol in all life stages, adolescence, young adulthood, and adulthood, compared with controls (*p* value < 0.05).

**Table 1:** Distribution of baseline characteristics for cases and controls: the EpiGEICAM study.

Characteristics	Total (*N* = 1578)	Controls (*N* = 799)	Cases (*N* = 779)	*P* value^a^
Alcohol consumption, g/day, mean (±SD)				
Adolescence	2.8 ± 5.7	2.4 (±4.0)	3.3 (±7.0)	**<0.01**
Young adulthood	5.4 ± 8.4	4.8 (±6.6)	6.1 (±9.9)	**<0.01**
Adulthood	6.8 ± 10.0	6.0 ± (8.3)	7.6 (±11.4)	**<0.01**
Age, years, mean (±SD)	49 ± 9	49 (±9)	49 (±9)	0.87
Body mass index, Kg/m^2^, mean (±SD)	25.2 ± 4.6	25.1 (±4.4)	25.3 (±4.7)	0.42
Postmenopausal, *n* (%)	653 (41.4)	352 (44.1)	301 (38.6)	**0.03**
Educational level, *n* (%)				**0.05**
Primary school or less	239 (15.2)	116 (14.5)	123 (15.8)	
Secondary school	815 (51.7)	395 (49.4)	420 (53.9)	
University graduate or higher	524 (33.2)	288 (36.1)	236 (30.3)	
Smoking status, *n* (%)				0.35
Never smoker	568 (36.0)	281 (35.2)	287 (36.9)	
Ex-Smoker (≥6 months)	465 (29.5)	233 (29.2)	231 (29.7)	
Smoker (or ex-smoker <than 6 months)	545 (34.5)	285 (35.6)	260 (33.4)	
Physical activity, *n* (%)				0.07
Sedentary/lightly active	541 (34.2)	247 (31.0)	292 (37.6)	
Moderately active	598 (37.9)	326 (40.8)	273 (35.0)	
Active/very active	439 (27.8)	226 (28.2)	214 (27.4)	
Calories, kcal/day, mean (±SD)	1952 (±633)	1906 (±632)	2000 (±622)	**<0.01**
Adherence to the Mediterranean dietary pattern, score 1–10, mean (±SD)	5.9 (±1.4)	6.0 (±1.4)	5.8 ± (1.3)	0.06
Age at menarche, years, mean (±SD)	12 (±1.5)	12 (±1.5)	12 (±1.5)	0.11
Age at first birth, years, mean (±SD)	26 (±5.0)	26 (±5.0)	27 (±5.0)	0.08
Number of children, mean (±SD)	1.6 (±1.2)	1.6 (±1.2)	1.5 (±1.1)	0.22
Chronic diseases, *n* (%)	635 (40.2)	341 (42.7)	294 (37.7)	**0.05**
Previous benign breast problems, *n* (%)	316 (20.0)	152 (19.0)	164 (21.1)	0.31
Family history of breast cancer, *n* (%)	358 (22.7)	153 (19.2)	205 (26.3)	**<0.01**
Hormone replacement therapy use, *n* (%)	161 (10.2)	77 (9.6)	84 (10.8)	0.43

Data correspond to observed values for age, number of children, menopausal status, chronic diseases, family history of breast cancer and alcohol consumption, and to imputed values for the remaining variables in the table.

Bold values indicate statistical significance *p* ≤ 0.05.

*SD* standard deviation

^a^*P* value resulting from Pearson Chi-Square test (categorical variables with no missing values), from Student’s *t* test (continuous variables with no missing values), and from logistic regression models (variables with imputed values).

When exploring the association between categories of alcohol consumption and the risk of BC in each of the life stages (Table 2), consumption during adolescence and adulthood were independently related to BC risk. Compared to those with less alcohol consumption (<5 g/day), women consuming ≥15 g/day in adolescence (OR 2.46; 95% CI 1.04, 5.86; *P* for trend 0.038) and in adulthood (in the last 5 years) (OR 1.93; 95% CI 1.13, 3.29; *P* for trend 0.029) had about twice the risk of BC, regardless of consumption during the other two corresponding life stages. However, consumption during young adulthood was not associated with a major BC risk when adjusting for alcohol consumption during adolescence and adulthood (OR 0.87; 95% CI 0.48, 1.58; *P* for trend 0.932) (Table 2).

**Table 2:** Association between alcohol consumption in each life stage and breast cancer occurrence, estimated from three models including different sets of adjusting variables.

Total alcohol consumption (g/day)	Co.	Ca.	Model 1^a^ OR (95% CI)	Model 2^b^ OR (95% CI)	Model 3^c^ OR (95% CI)
*Adolescence* (*n* = 1314; 657 paired case–control)					
<5	560	526	1 (ref.)	1 (ref.)	1 (ref.)
5–<15	82	103	1.36 (1.00. 1.87)	1.37 (0.96. 1.95)	1.31 (0.89. 1.94)
≥15	15	28	2.17 (1.11. 4.26)	2.63 (1.22. 5.64)	2.46 (1.04. 5.86)
*P* for trend			0.011	0.006	0.038
*Young adulthood* (*n* = 1410; 705 paired case–control)					
<5	470	431	1 (ref.)	1 (ref.)	1 (ref.)
5–<15	184	200	1.22 (0.95. 1.56)	1.35 (1.02. 1.80)	1.13 (0.80. 1.59)
≥15	51	74	1.68 (1.12. 2.51)	1.66 (1.05. 2.60)	0.87 (0.48. 1.58)
*P* for trend			0.002	0.006	0.932
*Adulthood* (*n* = 1448; 724 paired case–control)					
<5	431	414	1 (ref.)	1 (ref.)	1 (ref.)
5–<15	221	198	0.96 (0.75. 1.23)	1.03 (0.78. 1.35)	0.96 (0.70. 1.32)
≥15	72	112	1.80 (1.25. 2.60)	2.09 (1.37. 3.20)	1.93 (1.13. 3.29)
*P* for trend			0.002	0.004	0.030

To test for linear trends across increasing categories of alcohol consumption, the median concentration within each category was included and treated as a continuous variable in the model.

*Ca* cases, *Co* controls, *OR* odds ratio, *CI* confidence interval.

^a^Adjusted for age at the time of recruitment and hospital.

^b^Additionally adjusted for BMI, menopausal status, an interaction term between BMI and menopausal status, calories, age at menarche, number of children, age at first child, smoking status, educational level, chronic diseases, hormone replacement therapy use, previous benign breast lesions, family history of breast cancer, physical activity, adherence to the Mediterranean dietary pattern.

^c^Additionally adjusted for alcohol consumption during the other stages of life (*n* = 1.278).

**Fig. 1:**
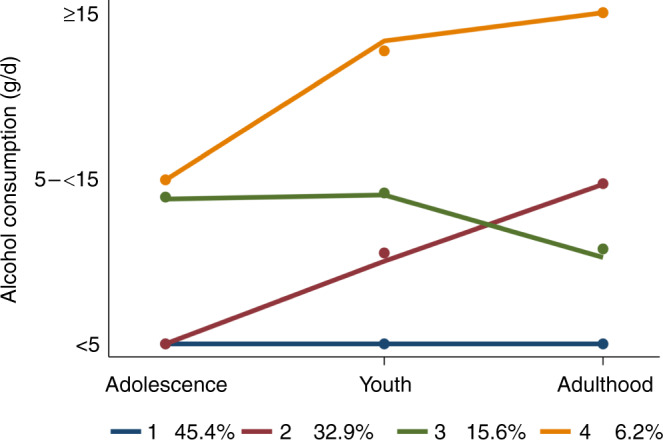
GBTM was used to identify different alcohol consumption trajectories throughout the life of those women with alcohol consumption data (categorised into low, moderate, and high according to the cut off points: <5, 5–<15 and ≥15 g/day; *X* axis) in each of the life stages: adolescence (12–19 yr.), young adulthood (20–29 yr.) and adulthood (≥30 yr.); *Y* axis. The percentages assigned to each trajectory represent the percentage of women out of the total sample (*N* = 1578) following that trajectory of alcohol consumption.

The best model identified by the GBTM involved four trajectories of alcohol consumption over the women´s lifetime (Fig. 1, Table S1), consistently when using the entire sample, as well as, when only using controls (Fig. S1). The first trajectory (followed by 45% of participants) comprised women who had consumed less than 5 g/d of alcohol throughout life. The second trajectory (33%) included those who progressively moved from a low alcohol consumption in adolescence (<5 g/day) to a moderate consumption in adulthood (5 to <15 g/day), never having a high consumption. Women in the third trajectory (16%) kept a moderate consumption throughout their lives. Finally, women in the fourth trajectory (6%) moved from a moderate alcohol consumption in adolescence (5 to <15 g/day) to a higher consumption in young adulthood (≥15 g/day).

When comparing the characteristics of control women following the four alcohol trajectories shown in Table S3, apart from alcohol intake, there were differences in age, tobacco consumption, age at first birth and history of benign breast lesions. Women in the fourth trajectory were more frequently smokers, had their first child at a later age and reported more frequently a history of benign breast lesion.

Table 3 shows the association between these lifetime trajectories of alcohol consumption and BC risk in the total sample and by menopausal status and BMI. Overall, a growing positive association between sequential trajectories and BC was observed. That is, comparing with the first trajectory, the fourth trajectory was the most strongly associated with BC (OR 2.19; 95% CI 1.27, 3.77), followed by the third trajectory (OR 1.44; 95% CI 0.96, 2.16) and ultimately by the second one (OR 1.17; 95% CI 0.86, 1.58). Thus, women with moderate consumption in adolescence that increased to a higher consumption in adulthood (fourth trajectory) had twice the risk of BC compared with women consuming less than 5 g/day of alcohol throughout life (first trajectory). When we analysed by menopausal status, although the trend of the associations remained, their magnitude with respect to the global ones, became greater in the group of postmenopausal women and diminished in premenopausal women.

**Table 3:** Association between alcohol consumption trajectories and breast cancer occurrence: total sample, by menopausal status and by body mass index in postmenopausal women.

Alcohol consumption trajectories	Controls	Cases	Model 1^a^ OR (95% CI)	Model 2^b^ OR (95% CI)
*Total* (*N* = 1278; 639 paired Ca/Co)				
Trajectory 1	304	280	1 (ref.)	1 (ref.)
Trajectory 2	228	216	1.05 (0.80. 1.37)	1.17 (0.86. 1.58)
Trajectory 3	70	86	1.38 (0.96. 1.97)	1.44 (0.96. 2.16)
Trajectory 4	37	57	1.79 (1.12. 2.88)	2.19 (1.27. 3.77)
*Premenopausal* (*n* = 925)				
Trajectory 1	217	212	1 (ref.)	1 (ref.)
Trajectory 2	151	160	1.07 (0.80. 1.43)	1.11 (0.81. 1.52)
Trajectory 3	53	68	1.35 (0.89. 2.03)	1.36 (0.88. 2.10)
Trajectory 4	26	38	1.48 (0.86. 2.52)	1.46 (0.82. 2.59)
*Postmenopausal* (*n* = 653)				
Trajectory 1	186	141	1 (ref.)	1 (ref.)
Trajectory 2	117	98	1.12 (0.79. 1.58)	1.22 (0.83. 1.79)
Trajectory 3	32	38	1.66 (0.98. 2.82)	1.79 (1.02. 3.15)
Trajectory 4	17	24	1.92 (0.99. 3.72)	2.33 (1.11. 4.93)
*Body mass index* < 25 kg/m^2^ (*n* = 263)^c^				
Trajectory 1	74	49	1 (ref.)	1 (ref.)
Trajectory 2	51	39	1.14 (0.66. 1.98)	1.75 (0.90. 3.38)
Trajectory 3	14	13	1.56 (0.65. 3.73)	1.79 (0.68. 4.69)
Trajectory 4	10	13	1.98 (0.80. 4.89)	3.28 (1.14. 9.39)
Body mass index ≥ 25 kg/m^2^ (*n* = 322)*				
Trajectory 1	93	75	1 (ref.)	1 (ref.)
Trajectory 2	54	49	1.16 (0.71. 1.91)	1.16 (0.65. 2.05)
Trajectory 3	16	18	1.36 (0.64. 2.86)	1.42 (0.62. 3.26)
Trajectory 4	7	10	1.80 (0.65. 5.00)	1.94 (0.60. 6.25)

*OR* odds ratio, *CI* confidence interval.

^a^Adjusted for age at the time of recruitment and hospital.

^b^Additionally adjusted for BMI, menopausal status, an interaction term between BMI and menopausal status, calories, age at menarche, number of children, age at first child, smoking status, educational level, chronic diseases, hormone replacement therapy use, previous benign breast lesions, family history of breast cancer, physical activity, adherence to the Mediterranean dietary pattern.

^c^Women with imputed BMI were excluded for this analysis (*n* = 68).

In postmenopausal women, trajectories were associated with BC with fully adjusted ORs of 2.33 (95% CI 1.11, 4.93), 1.79 (95% CI 1.02, 3.15) and 1.22 (95% CI 0.83, 1.79), respectively, for the fourth, third and second trajectories compared with the first one. In premenopausal women, although none of the trajectories reached statistical significance, the trajectories showed similar magnitudes of effect estimates. When postmenopausal women were separately analysed according to BMI, the magnitude of the effect estimates were significantly greater in slender postmenopausal women, even though the same trend was observed in both BMI groups. Postmenopausal women with a BMI < 25 in the fourth alcohol trajectory had about three times higher BC risk (95% CI 1.14, 9.39) compared with those in the first trajectory. However, the test for interaction between alcohol and BMI, as well as alcohol and menopausal status were not statistically significant (*P* value from likelihood-ratio test >0.1).

Finally, when examining the association between alcohol trajectories and BC risk by pathological subtype, no significant differences (Wald test *p* value >0.05) were seen, except for the luminal HER2 negative subtype (ER+/PR+ and HER2−) (OR, alcohol trajectory 4 vs 1, 1.73; 95% CI 1.07, 2,82). Estimations for the other two subtypes failed to attain statistical significance probably due to the limited number of cases (Tables S2 and S4).

## Discussion

In this case–control study of 1578 pre-and postmenopausal women, some main observations about the alcohol trajectories identified should be emphasised: (1) half of the participants reported no or very mild consumption of alcohol throughout life (2) those women who already drank during adolescence and young adulthood, continued to drink similarly or more during adulthood and, (3) when examined separately, ≥15 g/day of alcohol consumption at adolescence and in adulthood (in the last 5 years) were both strongly and independently associated with BC risk. Likewise, a growing positive association between sequential trajectories and BC risk was consistently observed, particularly in postmenopausal and for the luminal HER2 negative subtype (70% of the cases).

Taking none or very low alcohol consumption throughout life as reference, the fourth trajectory (those who moved from a moderate alcohol consumption in adolescence to a higher consumption in adulthood, never having had a low alcohol consumption) was always the most strongly associated with BC, followed by the third trajectory (those who kept a moderate consumption throughout their lives) and ultimately by the second trajectory (those who gradually moved from a low alcohol consumption to a moderate consumption in adulthood, never having a high consumption). These results suggest that the greater the alcohol consumption accumulated throughout life, the greater the risk of BC; but also, that alcohol consumption during adolescence may particularly influence BC risk. In fact, the BC risk corresponding to the third trajectory was greater than that for the second trajectory; and whereas this trajectory 2 increases from a low alcohol consumption in adolescence to a moderate consumption in young and late adulthood, the trajectory 3 starts with a moderate consumption in adolescence, maintained during young adulthood, and goes to a lower consumption in adulthood. In view of this, alcohol consumption in early ages could acquire more weight than consumptions later in life in relation to BC risk later in life. Despite the caution required due to the 95% CIs overlap, this interpretation is reinforced by the results presented in Table 2; when alcohol consumption was independently examined at each life stage, ≥15 g/day of alcohol consumption in adolescence which was strongly associated with BC risk, followed by this consumption during adulthood (in past 5-year period). These findings add support to the importance of exposure between menarche and the first pregnancy in breast cancer development [9] but also the importance of considering the totality of a woman’s exposure to alcohol over her lifetime as the best measure, rather than those from one specific periods [32].

Some previous studies exploring whether intake during specific periods influenced BC risk, also reported similar and independent contributions of alcohol consumption during the early adult years and later adult years to BC risk [32–36]. The strongest methodologically was a prospective observational study of 105,986 women enrolled in the Nurses’ Health Study (NSH) followed from 1980 until 2008 with early adults and eight updated alcohol assessments during this period. An observed association between even low levels of alcohol consumption and BC risk was observed. While the most relevant measure was cumulative average alcohol consumption over long periods both drinking earlier (between the ages of 18–40) and later (after the age of 40) in adult life were independently associated with BC risk [32]. In other investigations, an increased BC risk was evident only for those who consumed alcohol at young ages [37, 38], while others found that early alcohol consumption was not an important determinant of risk, or even that alcohol consumption later in life was the greatest influence on BC risk [39–41].

It is worth noting that in these studies the timing of alcohol consumption was evaluated using different chronological age cut-offs and mixing exposure both before and after the first pregnancy. These discrepancies may help explain the inconsistent results on the impact of alcohol consumption in early adult life. Thus, in these studies, young ages and early consumption have been defined as less than 30 yrs. [35, 37, 41], between 15 and 20 yrs. [39], between 18 and 35 yrs. [36] or between 18 and 40 [32], to give but some examples. However, when alcohol consumption before and after the first pregnancy was explicitly addressed, alcohol consumption prior to the first full-time pregnancy seems to be more consistently associated with BC risk [33, 34].

A breast with undifferentiated structures has a high rate of cell proliferation and is more predisposed to undergo malignant transformation. Thus, the time period from menarche to the first pregnancy is a window of time when breast tissue seems to be particularly susceptible to carcinogens and neoplastic transformation [7]. It has been observed in rat models that the tumorigenic response is maximal when the carcinogen is administered during critical adolescence period, in which the mammary gland is undifferentiated and highly proliferating. Administration of carcinogens to pregnant or parous rats, on the other hand, fails to elicit a tumorigenic response, explained by the complete development of the gland attained during pregnancy [42].

The strong association between alcohol consumption in adolescence and BC risk observed in our study might be understood in the light of these data supporting the importance of exposure between menarche and the first pregnancy in BC development. On the other hand, the lack of association between alcohol consumption in young adulthood (20–29 yrs.) and BC could be explained because most women (~80%) gave birth during this period of life (stage of life), thus being less vulnerable to the carcinogenicity of alcohol than in the previous life stage(s) [9]. Having information on alcohol consumption both before and after giving birth would have been of interest in order to more precisely define these risk periods.

Lifetime exposure to oestrogens appears to be one mechanism underlying the association between alcohol and all types of mammary cancers, hormone-dependent and hormone-independent [43, 44]. Several studies have shown positive correlations between alcohol intake and plasma or urinary oestrogen levels [45–49]. This increased oestrogen level in women consuming alcohol is hypothesised to be due either to a decrease in the metabolic clearance of oestrogens or to increased secretion [50, 51]. The apparent carcinogenicity of oestrogens is attributed to receptor-mediated stimulation of cellular proliferation, that could result in-turn in accumulation of genetic damage and stimulation of the synthesis of growth factors that act on the mammary epithelial cells [42, 52].

Alcohol may also affect BC risk by acting as a co-carcinogen, improving the permeability of membranes to carcinogens, inhibiting their detoxification, and activating procarcinogens [53]. Alcohol also seems to influence the disposition and function of essential nutrients or dietary factors considered to be cancer protective, through the modification of folate status or a decrease of concentrations of B-carotene, lutein/zeaxanthin, and vitamin C [54].

In this study, the stronger effect observed in postmenopausal women and particularly in those no overweight or obese (although the interactions were not statistically significant), could be partly explained by the oestrogenic effect of alcohol, that might be more important in postmenopausal women, in whom the ovary is not functioning, and even more important in those with less amount of body fat, since fat is the main source of oestrogens after menopause. However, differences across menopausal status could likely be justified by age, since the older, the more accumulated exposure. On the other hand, evidence suggests that early onset of BC may be biologically and possibly aetiologically distinct from BC arising in older women [55] as well as, women diagnosed with BC at young ages may be more affected by genetic susceptibility than by environmental factors such as alcohol consumption [56]. Finally, higher circulating oestrogen levels has also been proposed as the mechanism to explain why obesity (which is associated with abnormally high expression of the enzyme aromatase in the breast and increased local oestrogen production) increases the risk of postmenopausal BC [47, 57, 58].

When we examined the alcohol trajectories by pathological BC subtype, a significant association was observed for the luminal HER2 negative subtype (70% of the cases), while associations for the other two subtypes failed to attain statistical significance. However, the small sample size in some categories limits the capacity of making firm conclusions. Previously, the European Prospective Investigation into Cancer and Nutrition (EPIC) study did not observe heterogeneity in associations of alcohol and BC molecular subtype [34]. By contrast, alcohol consumption in prospective analysis of the nurses’ health study was associated with increased risk of luminal A and HER2-type BC, but not significantly associated with other subtypes after 26 years of follow-up. The authors suggested the notion of different etiologies across subtypes [59].

Some limitations of the study must be recognised. One of major limitations of this study is differential recall bias between cases and controls. This is suggested by much higher ORs for alcohol consumption in this study compared with that reported in the prospective studies. However, the similarities between cases and controls in terms of age, lifestyles, and environment, could reduce this likelihood. Also, the reliability and validity of reports of alcohol consumption in case–control studies are generally considered as acceptable [60]. Concerns about selection and information bias are legitimate as in any case–control study, but low rates of non-participation are reassuring. The baseline characteristics were similar between the women included in the study (*n* = 1578) and those excluded for having missing data in relation to alcohol consumption (*n* = 456), except for the educational level and smoking. The higher educational level of the women included in the study (compared with those excluded) may have affected the representativeness of the sample. On the other hand, this higher educational level allows for a better understanding of the questionnaire and for a higher accuracy in their self-reported information as well as may have even reduced potential confounding by socioeconomic status and other potential factors.

Controls were selected by the case from non-blood relatives, friends, neighbours, or work colleagues. This control identification has the potential to make lifestyle, including alcohol consumption patterns, more similar. Nonetheless, this approach is advantageous in terms of reducing biases, including being more homogeneous in their reproductive patterns, less potential differences in understanding and reporting their exposures as well as more homogeneity in their exposure to other environmental risk factors associated with social class. This approach, however, can lead to loss of efficiency due to overmatching and loss of representativeness of the alcohol consumption. On the other hand, this could serve to make the associations reported a conservative estimate of the true magnitude.

The fact that nearly half of the women recruited had a very low consumption of alcohol may have conditioned the statistical power of our analyses, particularly when assessing BC risk by tumour subtypes. Thus, the sample size in the fourth category (heaviest drinkers) was quite limited and therefore stratification by BMI and BC subtypes could have provided unstable estimates and caution in the interpretation is necessary. Likewise, although in this sample of Spanish women the total consumption of alcohol comes mainly from beer and wine, sample size limited the possibility of evaluating specific alcoholic beverages. Finally, women (whose age ranges were 30–60 years) were asked for alcohol consumption in the last 5 years, which applies to a different age for each woman. However, we assumed that alcohol habits likely remain similar throughout adulthood and consequently this reported consumption was considered as the average consumption during adulthood.

Using the GBTM, women were classified into different trajectory groups (mutually exclusive), and in this way allows us to sift through different patterns of alcohol consumption during life course. This novel approach has the advantage over traditional analysis in that this approach captures long term effects and critical or sensitive periods throughout life [13, 14]. In that sense, these findings may give a more reliable picture of how alcohol consumption across the life span affects the risk of developing BC than those from studies that measure cumulative exposure throughout life or only in a specific life period. The study has a multicentric design with a high participation rate (82%) and a large sample size, including case–control pairs matched by region and age. Likewise, detailed consumption of different alcoholic beverages was carefully collected, as well as other relevant variables.

The topic is of a major public health relevance because of the high prevalence of both exposure and outcome in our country. While in the past women used to consume alcohol less often, and in lower amounts as compared to men, currently, consumption is almost equated between both sexes in many countries. Europe is the heaviest drinking region in the world, for both men and women, and 14 years of age is now the average age when boys and girls start consuming alcohol [61]. In the most recent European school survey project on alcohol [62], about 50% of 15–16-year old female students reported having drunk alcohol at least once during the past month (the frequency of drinking alcohol was 5.4 occasions per month on average), and 35% of them also reported binge drinking, which was quite similar to their male counterparts. These statistics are similar in Spain [63]. Consequently, from today´s habits of alcohol consumption among teenagers, a future increase in the incidence of alcohol-related BC can be expected and shifting the focus of BC prevention to this age group is urgently needed.

## Conclusion

The study’s findings indicate that the more alcohol is consumed throughout life, the greater the risk of BC, especially in postmenopausal women. Alcohol consumption during adolescence could exert a great influence on BC risk. Likewise, alcohol consumption trajectories displayed that those women who started consuming alcohol in adolescence (≥5 g/day), continue to consume similar amounts or more during adulthood. Therefore, BC prevention strategies should target not only middle aged and older women, but also include adolescent girls and young women, whose current alcohol consumption is increasing alarmingly.

## Supplementary information

Supplemental Material

Reproducibility checklist

Strobe chechlist

## Data Availability

The data are available upon request from researchers interested in replicating the study.
