# Supplementary material for: Trajectories of alcohol consumption during life and the risk of developing breast cancer
Source: Br J Cancer. 2021 Sep 6;125(8):1168–76. doi: 10.1038/s41416-021-01492-w (PMC8505448; doi:10.1038/s41416-021-01492-w)
Supplement: Supplementary file 1 — Supplemental Material [file 41416_2021_1492_MOESM1_ESM.docx]

**Supplemental material**

Title:

**LIFETIME TRAJECTORIES OF ALCOHOL CONSUMPTION AND BREAST CANCER RISK**

| **Table S1.** Parameter estimates of the group-based trajectory modeling | | | | | |
| --- | --- | --- | --- | --- | --- |
| Trajectory | n | Co. | Ca. | APP | OCC |
| 1 | 756 | 403 | 353 | 0.9467159 | 30.03523 |
| 2 | 526 | 268 | 258 | 0.9056331 | 27.51365 |
| 3 | 191 | 85 | 106 | 0.9954383 | 2105.608 |
| 4 | 105 | 43 | 62 | 0.8821724 | 137.5464 |
| **APP**: Average posterior probability (model adequacy when APP ≥ 0.70)  **OCC**: Odds of correct classification (high assignment accuracy when OCC > 5) | | | | | |

| **Table S2.** Cases by each tumor subtype and controls according to the alcohol consumption trajectories | | | | | |
| --- | --- | --- | --- | --- | --- |
| **Alcohol consumption** | **Controls** | **ER+/PR+ and HER2-** | **HER2+** | **ER-, PR- and HER2-** | **Total** |
| **Trajectory 1** | 403 | 242 | 63 | 47 | 755 |
| **Trajectory 2** | 268 | 190 | 47 | 21 | 526 |
| **Trajectory 3** | 85 | 71 | 23 | 12 | 191 |
| **Trajectory 4** | 43 | 43 | 11 | 7 | 104 |
| Total | **799** | **546** | **144** | **87** | **1,576** |

| **Table S3.** Distribution of baseline characteristics according to alcohol consumption trajectories in controls (n=799) | | | | | |
| --- | --- | --- | --- | --- | --- |
|  | **Trajectory 1** | **Trajectory 2** | **Trajectory 3** | **Trajectory 4** | **P value*** |
| **Characteristics** | **(n=403)** | **(n=268)** | **(n=85)** | **(n=43)** |  |
| Alcohol consumption, g/day,  mean (±SD) |  |  |  |  |  |
| Adolescence | 0.9 (±1.2) | 1.5 (±1.4) | 8.8 (±3.5) | 9.7 (±9.6) | **<0.01** |
| Young adulthood | 1.3 (±1.4) | 6.0 (±4.7) | 9.9 (±10.0) | 24.9 (±15.1) | **<0.01** |
| Adulthood | 1.3 (±1.4) | 10.3 (±8.5) | 5.90 (±5.30) | 29.9 (±16.2) | **<0.01** |
| Age, years, mean (±SD) | 49.6 (±9.3) | 49.7 (±8.8) | 47.4 (±9.8) | 49.1 (±8.7) | **<0.01** |
| Body mass index, kg/m^2^, mean (±SD) | 25.4 (±4.6) | 24.9 (±3.9) | 25.0 (±4.4) | 23.9 (±3.2) | 0.08 |
| Postmenopausal, n (%) | 186 (46.2) | 117 (43.6) | 32 (37.7) | 17 (39.5) | 0.47 |
| Educational level, n (%) |  |  |  |  | 0.16 |
| Primary school or less | 70 (17.4) | 29 (10.8) | 13 (15.3) | 4 (9.3) |  |
| Secondary school | 199 (49.4) | 139 (51.9) | 38 (44.7) | 19 (44.2) |  |
| University graduate or higher | 134 (33.3) | 100 (37.3) | 34 (40.0) | 20 (46.5) |  |
| Smoking status, n (%) |  |  |  |  | **<0.01** |
| Never smoker | 182 (45.2) | 70 (26.1) | 23 (27.1) | 6 (14.0) |  |
| Ex-Smoker (≥6 months) | 97 (24.2) | 93 (34.7) | 33 (38.8) | 10 (23.3) |  |
| Smoker (or ex-smoker < than 6 months) | 124 (30.7) | 105 (39.2) | 29 (34.1) | 27 (62.8) |  |
| Physical activity, n (%) |  |  |  |  | 0.62 |
| Sedentary/lightly active | 124 (30.9) | 84 (31.3) | 28 (33.2) | 11 (25.6) |  |
| Moderately active | 156 (38.7) | 119 (44.5) | 33 (38.3) | 18 (41.9) |  |
| Quite active/Very active | 123 (30.4) | 65 (24.2) | 24 (28.5) | 14 (32.5) |  |
| Calories. kcal/day, mean (±SD) | 1911 (±672) | 1893 (±622) | 1826 (±573) | 2.101 (±540) | 0.16 |
| Adherence to the Mediterranean dietary pattern. score 1-10, mean (±SD) | 5.9 (±1.5) | 6.0 (±1.4) | 5.7 (±1.2) | 6.3 (±1.3) | 0.21 |
| Age at menarche, years, mean (±SD) | 12.3 (±1.4) | 12.4 (±1.6) | 12.7 (±1.6) | 12.7 (±1.5) | 0.23 |
| Age at first birth, years, mean (±SD) | 25.7 (±4.2) | 26.5±4.6) | 26.8 (±4.1) | 27.0 (±3.5) | **0.02** |
| Number of children. mean (±SD) | 1.6 (±1.2) | 1.6 (±1.2) | 1.5 (±1.2) | 1.3 (±1.0) | 0.14 |
| Chronic diseases, n (%) | 179 (44.5) | 116 (43.2) | 34 (40.2) | 12 (27.9) | 0.19 |
| Previous benign breast problems. n (%) | 80 (19.9) | 54 (20.2) | 7 (8.2) | 11 (25.6) | **0.05** |
| Family history of breast cancer, n (%) | 72 (17.9) | 48 (17.9) | 21 (24.7) | 12 (27.9) | 0.21 |
| Hormone replacement therapy use, n (%) | 42 (10.5) | 27 (10.0) | 5 (6.1) | 2 (5.6) | 0.53 |
| Data correspond to observed values for age, number of children, menopausal status, chronic diseases, family history of breast cancer and alcohol consumption, and to imputed values for the remaining variables in the table.  SD = standard deviation  * P value resulting from Pearson Chi-Square test (categorical variables with no missing values), from Student's t test (continuous variables with no missing values), and from logistic regression models (variables with imputed values). | | | | | |

| **Table S4.** Adjusted relative-risk ratios (RRR) for the association between alcohol consumption trajectories and breast cancer occurrence by pathological tumor subtypes | | | | | |  |
| --- | --- | --- | --- | --- | --- | --- |
| **Trajectory** |  |  | **Model 1^1^**  **RRR (95% CI)** | **Model 2 ^2^**  **RRR (95% CI)** |  |  |
| **ER+/PR+ and HER2-** (n=546) | | | | |  |  |
| Trajectory 1 |  |  | 1 (ref.) | 1 (ref.) |  |  |
| Trajectory 2 |  |  | 1.18 (0.92. 1.50) | 1.22 (0.94. 1.58) |  |  |
| Trajectory 3 |  |  | 1.41 (0.99. 2.01) | 1.50 (1.03. 2.17) |  |  |
| Trajectory 4 |  |  | 1.65 (1.05. 2.60) | 1.73 (1.07. 2.82) |  |  |
| **HER2+** (n=144) | | | | |  |  |
| Trajectory 1 |  |  | 1 (ref.) | 1 (ref.) |  |  |
| Trajectory 2 |  |  | 1.11 (0.74. 1.67) | 1.17 (0.76. 1.78) |  |  |
| Trajectory 3 |  |  | 1.80 (1.05. 3.07) | 1.76 (1.01. 3.06) |  |  |
| Trajectory 4 |  |  | 1.61 (0.79. 3.29) | 1.59 (0.75. 3.35) |  |  |
| **ER-, PR- and HER2-** (n=87) | | | | |  |  |
| Trajectory 1 |  |  | 1 (ref.) | 1 (ref.) |  |  |
| Trajectory 2 |  |  | 0.69 (0.40. 1.18) | 0.71 (0.40. 1.25) |  |  |
| Trajectory 3 |  |  | 1.14 (0.58. 2.26) | 1.19 (0.58. 2.42) |  |  |
| Trajectory 4 |  |  | 1.45 (0.62. 3.42) | 1.50 (0.59. 3.83) |  |  |
| **Abbreviations: ER:** Estrogen receptor**; PR:** Progesterone receptor**; HER2:** Luminal human epidermal growth factor receptor 2  **1** Adjusted for age at the time of recruitment and hospital  **2** Additionally adjusted for BMI, menopausal status, an interaction term between BMI and menopausal status, calories, age at menarche, number of children, age at first child, smoking status, educational level, chronic diseases, hormone replacement therapy use, previous benign breast lesions, family history of breast cancer, physical activity, adherence to the Mediterranean dietary pattern | | | | |  |  |

| **Figure S1** Lifetime trajectories of total alcohol consumption separately in controls (n=799) |
| --- |
| 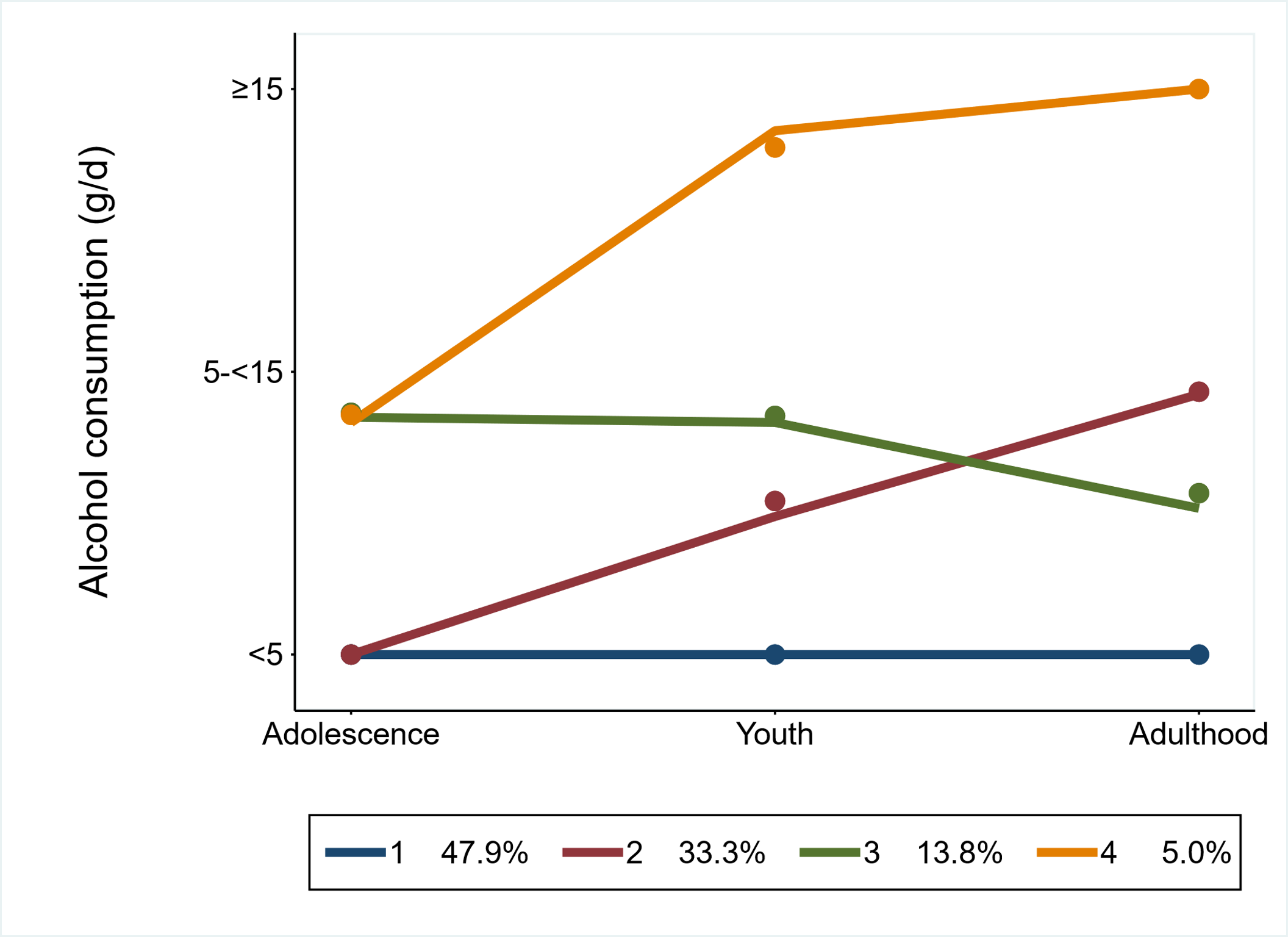 |
| GBTM was used to identify different alcohol consumption trajectories throughout the life of those women with alcohol consumption data (categorized into low, moderate and high according to the cut off points: <5, 5–<15 and ≥15 g/day; **X axis**) in each of the life stages: adolescence (12-19 yr.), young adulthood (20-29 yr.) and adulthood (≥30 yr.); **Y axis**. The percentages assigned to each trajectory represent the percentage of controls following that trajectory of alcohol consumption. |
